# Luteolin Inhibits Bovine Viral Diarrhea Virus Replication by Disrupting Viral Internalization and Replication and Interfering with the NF-κB/STAT3-NLRP3 Inflammasome Pathway

**DOI:** 10.3390/vetsci13010057

**Published:** 2026-01-07

**Authors:** Dongjie Cai, Qing Liu, Zifan Shen, Bin Tian, Jiabin Gao, Yulin Lin, Lanjing Ma, Ya Wang, Xiaoping Ma

**Affiliations:** 1 Key Laboratory of Animal Disease and Human Health of Sichuan Province, College of Veterinary Medicine, Sichuan Agricultural University, Huimin Road, Wenjiang District, Chengdu 611130, China; 15198037081@163.com (Q.L.); richard_shen_zifan@163.com (Z.S.); zhaoyq2728@163.com (Y.L.); malanjing2000@163.com (L.M.); wangyayang@126.com (Y.W.); mxp886@sicau.edu.cn (X.M.); 2 Institute of Preventive Veterinary Medicine, Sichuan Agricultural University, Chengdu 611130, China; 14730@sicau.edu.cn; 3 Baicheng Institute of Animal Husbandry, Baicheng 137000, China; m15204367066@163.com

**Keywords:** BVDV, luteolin, NF-κB/STAT3-NLRP3

## Abstract

Bovine viral diarrhea virus (BVDV) causes severe mucosal inflammation in cattle, and effective treatment options remain limited. Dysregulated activation of the NLRP3 inflammasome, driven by NF-κB and STAT3 signaling, may exacerbate disease pathogenesis, highlighting this axis as a potential therapeutic target. Although traditional Chinese medicine has shown promise in antiviral and anti-inflammatory applications, it remains unclear whether it can inhibit BVDV replication via the NF-κB/STAT3-NLRP3 pathway. The present study aimed to clarify the inhibitory effect of luteolin on bovine viral diarrhea virus (BVDV) replication, and to elucidate its underlying mechanisms from two perspectives: interference with viral internalization and replication processes, as well as regulation of the NF-κB/STAT3-NLRP3 inflammasome pathway. Collectively, this work intended to provide experimental evidence and theoretical support for the development of luteolin as a natural anti-BVDV agent. To this end, BVDV-infected MDBK cells were treated with gradient concentrations of luteolin, followed by quantification of viral load using qRT-PCR and Western blot assays. Meanwhile, the activation status of the NF-κB/STAT3-NLRP3 signaling pathway was evaluated via immunofluorescence staining and luciferase reporter gene assays. Our results demonstrate that luteolin exhibits potent dual antiviral activity against cytopathic BVDV-1m in MDBK (Madin-Darby Bovine Kidney) cells, effectively suppressing both viral replication and inflammatory responses. At non-cytotoxic concentrations, luteolin specifically inhibited the internalization and replication stages of the viral lifecycle, accompanied by reduced NS5B polymerase activity. Importantly, luteolin disrupted the NF-κB/STAT3-NLRP3 axis by suppressing phosphorylation of p65 (Ser536) and STAT3 (Ser727), downregulating NLRP3 and pro-caspase-1 expression, and inhibiting caspase-1 cleavage (p20) as well as maturation of IL-1β and IL-18. Consequently, it attenuated the overexpression of TNF-α and IL-8. To our knowledge, this is the first report of a single compound simultaneously targeting multiple stages of the BVDV lifecycle and counteracting NLRP3-mediated immunopathology, offering a strategic basis for developing flavonoid-based therapies against Flavivirus infections.

## Simple Summary

This study focuses on the inhibitory effect and underlying mechanisms of luteolin, a natural flavonoid compound, against bovine viral diarrhea virus (BVDV). Experiments on MDBK cells revealed that luteolin exhibits no cytotoxicity at concentrations below 20 μM and inhibits the replication of BVDV-1m strain in a dose-dependent manner. Its core mechanisms involve two aspects: first, it directly targets the internalization and replication stages of the viral life cycle, inhibits the activity of NS5B polymerase, and exerts no direct effect on viral attachment, release, or the viral particles themselves; second, it blocks the activation of the NF-κB/STAT3-NLRP3 signaling axis by inhibiting the phosphorylation of p65 (Ser536) and STAT3 (Ser727), downregulating the transcriptional expression of NLRP3 and pro-caspase-1, preventing the cleavage of caspase-1 into the p20 subunit, and inhibiting the maturation of IL-1β/IL-18, thereby reducing the excessive production of inflammatory mediators such as TNF-α and IL-8. This study is the first to confirm that luteolin possesses dual antiviral and anti-inflammatory activities, providing crucial experimental evidence and a theoretical basis for the development of flavonoid-based drugs against Flaviviridae viruses. Meanwhile, it points out that the clinical application of luteolin needs to address issues such as low bioavailability, potential immunosuppression, and viral drug resistance.

## 1. Introduction

Bovine viral diarrhea virus (BVDV), a member of the Flaviviridae family and Pestivirus genus, is a single-stranded, positive-sense RNA virus. Its genome consists of a large open reading frame flanked by 5′ and 3′ untranslated regions that are essential for viral replication [1]. BVDV is classified into two biotypes: cytopathogenic strains, which trigger apoptosis in infected cells in vitro, and non-cytopathogenic strains, capable of establishing persistent infections without apparent cytopathic effects [2]. As the causative agent of bovine viral diarrhea (BVD), this virus infects various domestic animals, including cattle, sheep, and pigs [3]. Clinical symptoms vary widely, ranging from acute manifestations—such as fever, diarrhea, nasal discharge, leukocytosis, and gastrointestinal erosions—to reproductive disorders like spontaneous abortion. A major epidemiological concern is persistent infection, wherein immunotolerant animals become lifelong carriers, continuously shedding the virus and maintaining transmission within herds [4]. Owing to its broad host range, diverse clinical presentations, and sustained transmission cycles, BVDV poses a substantial threat to livestock production worldwide. Existing control measures show limited effectiveness, largely due to the virus’s genetic variability and immune evasion mechanisms [5]. This therapeutic gap highlights the urgent need for novel antiviral approaches to mitigate BVDV pathogenesis and transmission.

Upon viral infection, pattern recognition receptors such as TLR4 activate NF-κB through the MyD88-dependent pathway, thereby promoting the expression of pro-inflammatory factors (e.g., TNF-α and IL-6) as well as the NLRP3 precursor [6,7]. Concurrently, STAT3 is activated by IL-10 signaling, undergoes phosphorylation, and translocates into the nucleus, where it suppresses IRF3 transcriptional activity and reduces IFN-β production [8]. Furthermore, the activated NLRP3 inflammasome cleaves the IAD domain of IRF3 via caspase-1, disrupting its interaction with MAVS and thereby inhibiting type I interferon (IFN-I) production. This highlights a synergistic mechanism between the NF-κB/STAT3-NLRP3 pathway and IFN-I suppression during viral infection. STAT3 not only amplifies NLRP3 priming signals through NF-κB but also directly binds to the IRF3 promoter to repress its expression [9]. In contrast, the acetate-GPR43 pathway counteracts this suppression by enhancing IFN-I production via the NLRP3-MAVS axis [10,11]. Upon viral infection, pattern recognition receptors such as TLR4 activate NF-κB through the MyD88-dependent pathway, thereby promoting the expression of pro-inflammatory factors (e.g., TNF-α and IL-6) as well as the NLRP3 precursor [6,7]. Concurrently, STAT3 is activated by IL-10 signaling, undergoes phosphorylation, and translocates into the nucleus, where it suppresses IRF3 transcriptional activity and reduces IFN-β production [8]. Furthermore, the activated NLRP3 inflammasome cleaves the IAD domain of IRF3 via caspase-1, disrupting its interaction with MAVS and thereby inhibiting type I interferon (IFN-I) production. This highlights a synergistic mechanism between the NF-κB/STAT3-NLRP3 pathway and IFN-I suppression during viral infection. STAT3 not only amplifies NLRP3 priming signals through NF-κB but also directly binds to the IRF3 promoter to repress its expression [9]. In contrast, the acetate-GPR43 pathway counteracts this suppression by enhancing IFN-I production via the NLRP3–MAVS axis [10,11]. Although STAT3 is known as a negative regulator of type I interferon signaling, targeting STAT3 with compounds such as capsaicin represents a promising small-molecule strategy to enhance antiviral host responses [12]. Similarly, the STAT3 inhibitor stattic has been shown to overcome bortezomib resistance in multiple myeloma through downregulation of PSMB6 [9]. Overall, targeting the NF-κB/STAT3-mediated inflammatory pathway holds significant therapeutic potential and constitutes a crucial strategy for the development of antiviral treatments.

Luteolin (Lut), a natural flavonoid characterized by a C6–C3–C6 skeleton and polyhydroxy groups, demonstrates multi-target antiviral potential. Recent studies indicate its broad-spectrum efficacy against various viruses, including coronaviruses [13,14,15], herpesviruses [16], hepatitis B virus [17], and several RNA viruses. Its mechanisms involve direct inhibition of viral proteins and modulation of host immune responses. For instance, luteolin interferes with SARS-CoV-2 spike protein binding to angiotensin-converting enzyme 2 (ACE2) [15], inhibits viral entry and replication [18], and suppresses SARS-CoV-2 spike-induced platelet activation [19]. It also exhibits synergistic effects in combination with magnesium, zinc, and vitamin C, suggesting a potential immunomodulatory strategy for COVID-19 management [20]. Additionally, luteolin inhibits the SARS-CoV-2 3CL protease (IC_50_ = 2.9 μM) and competitively binds porcine ACE2 (Kd = 71.6 μM) [21]. In the context of COVID-19, it downregulates the CAMK2A/CHOP/MAPK and NF-κB pathways, reducing IL-6 and IL-1β release, and mitigates cytokine storms by inhibiting JAK1/STAT3 phosphorylation [22]. Against herpesviruses, luteolin activates the cGAS–STING pathway and promotes IRF3 phosphorylation, thereby reducing viral load [16]. In hepatitis B virus infection, its combination with Schisandrin C synergistically suppresses HBV replication and enhances cGAS–STING activation in macrophages [17]. Furthermore, luteolin attenuates spike glycoprotein S1-induced inflammation in THP-1 cells via the ER stress-mediated calcium/CHOP/MAPK pathway, leading to decreased production of IL-6 and IL-1β [14]. Despite these extensive findings, the anti-BVDV activity of luteolin and its underlying mechanisms remain entirely unexplored.

Infection with bovine viral diarrhea virus (BVDV) activates substantial inflammatory pathways that contribute to viral pathogenesis. Studies indicate that viral proteins such as NS5B and Erns trigger the NF-κB signaling cascade, leading to NLRP3 inflammasome assembly and caspase-1-dependent maturation of proinflammatory cytokines, including IL-1β and IL-18 [23,24]. This coordinated dysregulation of inflammatory responses creates a permissive environment for viral persistence and tissue injury.

The present study aimed to clarify the inhibitory effect and underlying molecular mechanism of luteolin on bovine viral diarrhea virus (BVDV) replication from two perspectives: viral life cycle intervention and host cellular inflammatory response regulation. Specifically, we sought to identify its blocking effect on viral internalization and replication processes, as well as its regulatory function in the NF-κB/STAT3-NLRP3 inflammasome pathway, thereby providing theoretical and experimental support for the development of luteolin as a natural anti-BVDV agent. In the present study, using a cytopathic BVDV-1m clinical strain (JLBC-28) isolated from cattle in Jilin Province, we systematically evaluated the anti-BVDV activity of luteolin. Our results show that luteolin inhibits viral replication specifically during the internalization and replication stages, without exhibiting direct virucidal effects. Importantly, luteolin also attenuated BVDV-induced hyperactivation of both NF-κB and STAT3 signaling pathways, thereby suppressing NLRP3 inflammasome formation. This dual inhibition of viral replication and inflammatory cascades reveals a novel antiviral mechanism against BVDV, offering a strategic basis for the development of flavonoid-based therapeutics.

## 2. Materials and Methods

### 2.1. Cells, Virus, Reagents and Instruments

Madin–Darby bovine kidney (MDBK) cells were cultured in Dulbecco’s modified Eagle’s medium/Ham’s F-12 medium (DMEM/F12, 1:1; Gibco Life Technologies, Grand Island, NY, USA) supplemented with 10% heat-inactivated fetal bovine serum (FBS; Gibco-BRL, Carlsbad, CA, USA) and 1% penicillin–streptomycin (Thermo Fisher Scientific, Waltham, MA, USA). Cells were maintained at 37 °C in a humidified 5% CO_2_ atmosphere. The cytolytic BVDV-1m strain JLBC-28 was isolated from clinical serum samples of diarrheic cattle in Jilin Province, China, and propagated in MDBK cells. The viral titer was determined as 10^6.75^ TCID_50_/0.1 mL using the Reed–Muench method. Virus stocks were stored at −80 °C. Luteolin (product number: T1027; purity ≥ 98%) was purchased from TargetMol Chemicals Inc. (Shanghai, China). A 50 mM stock solution was prepared in dimethyl sulfoxide (DMSO; Sigma-Aldrich, St. Louis, MO, USA) and stored at −20 °C protected from light. Working concentrations were prepared by dilution in serum-free DMEM/F12 immediately before use.

Luteolin was purchased from TargetMol Chemicals Inc. (Shanghai, China) with a purity of 98%. The CCK-8 kit, RIPA cell lysis buffer, BCA protein assay kit, and protease inhibitor cocktail were obtained from Beyotime Biotechnology (Shanghai, China). DMSO was purchased from Solarbio Science & Technology Co., Ltd. (Beijing, China). The 2× Realab Green PCR Fastmixture (universal type) was supplied by Lanbolide Biotech Co., Ltd. (Beijing, China). GAPDH Mouse mAb (catalog No. AC002), HRP-conjugated Goat anti-Rabbit IgG (H+L) (catalog No. AS014), and HRP-conjugated Goat anti-Mouse IgG (H+L) (catalog No. AS003) were purchased from Abclonal Technology Co., Ltd. (Wuhan, China). The mouse-derived anti-BVDV-E2 antibody was prepared and preserved in our laboratory. The ECL chemiluminescence kit was obtained from Yeasen Biotechnology Co., Ltd. (Shanghai, China).

The instruments used in this study included micropipettes purchased from Eppendorf (Hamburg, Germany), ultra-clean workbenches purchased from Antai Company of Sujing Group (Suzhou, China), high-capacity high-speed refrigerated centrifuges, floor-standing constant temperature and refrigerated shakers, cell incubators and biosafety cabinets purchased from Thermo Fisher Scientific (Waltham, MA, USA), as well as constant voltage and constant current electrophoresis apparatus purchased from Beijing Liuyi Biotechnology Co., Ltd. (Beijing, China).

### 2.2. Viral Infection

The BVDV JLBC-28 strain was diluted in serum-free DMEM/F12 medium to achieve a multiplicity of infection (MOI) of 1. Confluent MDBK cell monolayers were incubated with the viral inoculum at 37 °C for 2 h. Following incubation, cells were washed three times with phosphate-buffered saline (PBS) to remove unabsorbed viral particles. Infected cells were maintained in DMEM/F12 supplemented with 2% fetal bovine serum (FBS) and 1% penicillin–streptomycin for designated time periods according to experimental protocols.

### 2.3. Cytotoxicity Assay

Cell viability was assessed using the Cell Counting Kit-8 (Beyotime, Shanghai, China). MDBK cells were seeded in 96-well plates at 1 × 10^5^ cells/mL (100 μL/well) and incubated for 24 h. After replacing medium with fresh DMEM/F12 containing luteolin (1–200 μM), cells were incubated for 48 h. CCK-8 solution (10 μL/well) was added, and the plates were incubated at 37 °C for 1.5 h. Absorbance was measured at 450 nm using a microplate reader (BioTek Instruments, Winooski, VT, USA).

### 2.4. RNA Extraction and Quantitative RT-PCR

Total RNA was isolated from MDBK cells using Trizol reagent (TransGen Biotech, Beijing, China) in accordance with the manufacturer’s instructions. Complementary DNA (cDNA) was synthesized from 1 µg of total RNA with the PrimeScript™ II 1st Strand cDNA Synthesis Kit (TaKaRa, Kusatsu, Japan). Quantitative real-time polymerase chain reaction (qRT-PCR) with SYBR Green intercalating dye was performed using Taq SYBR^®^ Green qPCR Premix. The following primers were used: BVDV 5′-UTR (forward primer 5′-ATGCCCACGTAGGACTAGCA-3′ and reverse primer 5′-TCAACTCCATGTGCCATGTAC-3′; 288 bp), NLRP3 (forward primer 5′-AAGAAGCTCTGGTTGGTCAGTTGC-3′ and reverse primer 5′-GGAATGGTTGGTGCTCAGGACAG-3′), TNF-α (forward primer 5′-CTGGCGGAGGAGGTGCTCTC-3′ and reverse primer 5′-GGAGGAAGGAGAAGAGGCTGAGG-3′), pro-caspase-1 (forward primer 5′-GCTTGCATCTTCAGGACCAGGAG-3′ and reverse primer 5′-CAACATCAGCTCCGTCTCTTCTGG-3′), IL-8 (forward primer 5′-TGCCTGTTGAACTGCGCCTTG-3′ and reverse primer 5′-AGTGCTTCCACATGTCCTCACATC-3′), GAPDH (forward primer 5′-GATTGTCAGCAATGCCTCCT-3′ and reverse primer 5′-GGTCATAAGTCCCTCCACGA-3′). The qRT-PCR amplification protocol was programmed as follows: initial denaturation at 95 °C for 30 s; 40 cycles of denaturation at 95 °C for 5 s and annealing/extension at 60 °C for 30 s. A melt curve analysis was then carried out, with the temperature increasing from 65 °C to 95 °C at a rate of 0.5 °C/s to verify primer specificity. Relative gene expression levels were calculated via the 2^−ΔΔCt^ method, with GAPDH serving as the internal reference gene for normalization. All experimental procedures were performed following established protocols, and each reaction was conducted in triplicate to ensure the reliability of results.

### 2.5. Western Blot Analysis

Protein samples were extracted from MDBK cells using RIPA lysis buffer supplemented with 1% protease inhibitor cocktail. Protein concentrations were quantified via BCA assay, and equal amounts (20–40 μg/lane) were separated by 10% SDS-PAGE. Proteins were transferred onto PVDF membranes (0.45 μm pore size) using a wet transfer system (200 mA, 90–120 min). Membranes were blocked with 5% skim milk in TBST (Tris-buffered saline with 0.1% Tween-20) for 1 h at room temperature, followed by overnight incubation at 4 °C with primary antibodies against target proteins. After three washes with TBST (10 min each), membranes were incubated with species-matched HRP-conjugated secondary antibodies for 1 h at room temperature. Protein bands were visualized using enhanced chemiluminescence (ECL) substrate and imaged under a chemiluminescence detection system.

### 2.6. Antiviral Activity Assessment

Luteolin was dissolved in DMSO to generate a 20 mM stock solution and serially diluted in PBS to final concentrations of 1, 5, 10, and 20 μM. Confluent MDBK cell monolayers (80–90% density) were infected with BVDV strain JLBC-28 at a multiplicity of infection (MOI) of 1. For dose-dependent assays, infected cells were treated with luteolin (1–20 μM) or DMSO for 48 h. To evaluate administration timing, three regimens were implemented: Pre-treatment: Cells incubated with 20 μM luteolin for 2 h prior to viral infection. Co-treatment: luteolin (20 μM) and virus added simultaneously. Post-treatment: luteolin (20 μM) applied 2 h post-infection. After 24 h incubation, cells were harvested for qRT-PCR and immunoblotting analysis.

### 2.7. Viral Lifecycle Stage Analysis

Direct virucidal effect: luteolin (20 μM) and BVDV (MOI = 1) were mixed 1:1 (*v*/*v*), incubated at 37 °C for 2 h, then purified through 20% sucrose cushion centrifugation (90,000× *g*, 4 °C, 1.5 h). Purified virions were used to infect MDBK cells. Attachment inhibition: Cells were pre-chilled at 4 °C, treated with 20 μM luteolin or DMSO for 1 h, then exposed to BVDV (MOI = 1) at 4 °C for 2 h. Unbound viruses were removed by PBS washing. Internalization assay: After viral adsorption (4 °C, 1 h), cells were washed and incubated with luteolin (20 μM) at 37 °C for 2 h. Non-internalized viruses were eliminated by PBS (pH 3.0) washing. Replication inhibition: Following viral entry (37 °C, 2 h), cells were treated with luteolin (20 μM) for 10 h at 37 °C. Release inhibition: At 10 h post-infection, luteolin (20 μM) was added for 2 h before supernatant collection.

### 2.8. Statistical Analysis

Statistical analyses were performed using GraphPad Prism 8.0 software (https://www.graphpad.com/, accessed on 28 October 2024). An unpaired Student *t*-test was used to assess the statistical significance of comparing two means. A one-way analysis of variance (ANOVA) test was used for dose-dependent experiments or multiple comparisons, followed by a post hoc test (Dunnett or Tukey test). Quantitative data in histograms are shown as means ± SD. Statistical relevance was evaluated using the following *p*-values: * *p* < 0.05, ** *p* < 0.01, *** *p* < 0.001.

## 3. Results

### 3.1. Dose and Time Dependent Inhibition of BVDV by Luteolin

**Figure 1:**
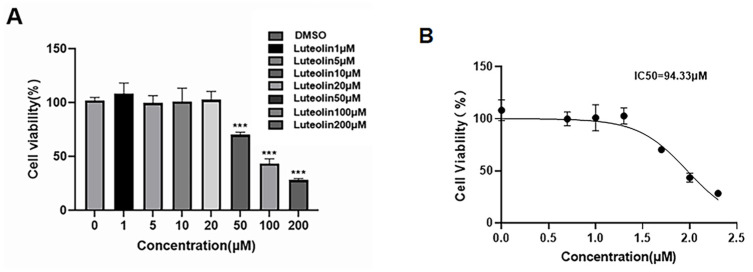
(**A**) Effects of different concentrations of luteolin on the activity of MDBK cells at 48 h. (**B**) Dose-response relationship of luteolin on MDBK cells. (The data are expressed as the mean ± standard deviation, *** *p* < 0.001).

To investigate whether luteolin exerts anti-bovine viral diarrhea virus (BVDV) activity, we first evaluated its biocompatibility in Madin-Darby bovine kidney (MDBK) cells. Cellular viability was measured to assess the status of cells treated with different concentrations of luteolin. The results showed that luteolin at concentrations of 1, 5, 10, or 20 μM did not induce obvious cytotoxicity in MDBK cells when compared with the dimethyl sulfoxide (DMSO) control group (Figure 1A). However, it significantly reduced cell viability over 50 μM (Figure 1). The dose-response relationship between the logarithm of luteolin concentration (X) and cell inhibition rate (Y) is shown in Figure 1B. The IC_50_ of luteolin was calculated as 94.33 μM. Therefore, 20 μM was established as the maximum non-toxic concentration for subsequent antiviral experiments.

**Figure 2:**
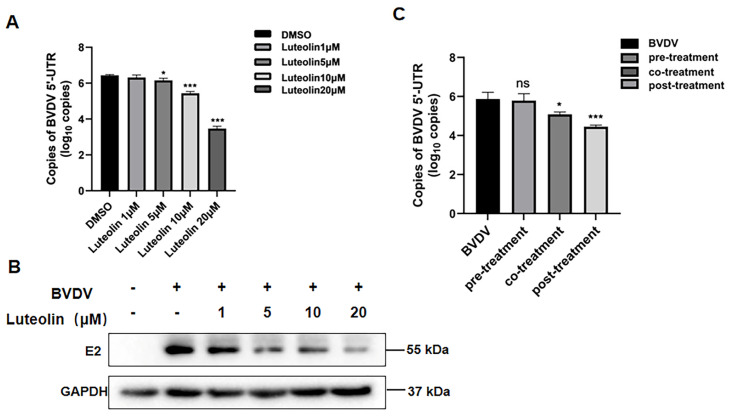
(**A**) Effect of different concentrations of luteolin on BVDV copy number. (**B**) Effect of different concentrations of luteolin on BVDV E2 protein level. (**C**) Effect of luteolin on BVDV by different treatments. (The data are expressed as the mean ± standard deviation, * *p* < 0.05, *** *p* < 0.001, ns: *p* > 0.05).

Then, MDBK cells were infected with BVDV and subsequently treated with luteolin at varying concentrations. The dose-dependent antiviral activity of luteolin was evaluated by quantifying viral RNA copy numbers via quantitative real-time polymerase chain reaction (qRT-PCR). When administered at 2 h post-infection (hpi), qRT-PCR analysis revealed that 5 μM luteolin significantly reduced viral RNA copies compared to the DMSO control (* *p* < 0.05), while 10 μM and 20 μM treatments caused marked reductions of viral load (*** *p* < 0.001) (Figure 2A), indicating luteolin exhibited potent dose-dependent suppression of BVDV replication in MDBK cells infected at 1 MOI. Furthermore, luteolin, at dose-responsive levels, inhibits the expression of BVDV viral structural protein E2, as confirmed by western blot analysis, with near-complete suppression observed at 20 μM (Figure 2B and Figure S1). The antiviral efficacy was critically dependent on treatment timing. Pre-treatment of cells prior to viral exposure showed no significant inhibition (ns, *p* > 0.05). In contrast, co-treatment (luteolin was added only during the period of viral infecting cells) significantly reduced viral RNA (* *p* < 0.05), while maximal suppression occurred with post-treatment (luteolin added at 2 hpi), achieving *** *p* < 0.001 reduction (Figure 2C). These results indicate that luteolin primarily targets post-entry stages of the BVDV lifecycle.

### 3.2. Effect of Luteolin on Internalization and Replication of BVDV

**Figure 3:**
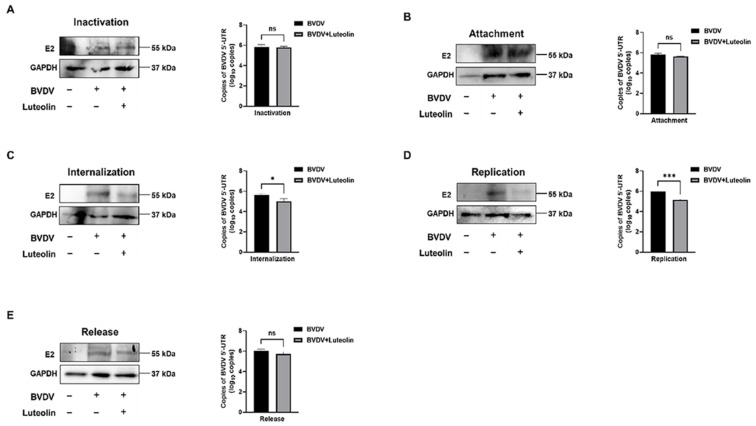
(**A**) Inactivation effect of luteolin on BVDV. (**B**) Effect of luteolin on attachment of BVDV. (**C**) Effect of luteolin on internalization of BVDV. (**D**) Effect of luteolin on replication of BVDV. (**E**) Effect of luteolin on the release of BVDV (The data are expressed as the mean ± standard deviation, *** *p* < 0.001, ns: *p* > 0.05, * *p* < 0.05).

To further define the inhibitory effect of luteolin on the replication life cycle of BVDV, the viral life cycle was dissected into distinct stages through precisely controlled experimental manipulations. Western blotting and RT-qPCR were employed to analyze BVDV-infected MDBK cells treated with luteolin (20 μM). We first assessed whether luteolin exhibited direct virucidal activity against BVDV particles. Western blotting and RT-qPCR revealed no significant reduction in BVDV E2 protein expression or viral 5′-UTR mRNA levels in luteolin-treated groups compared to BVDV-infected controls (*p* > 0.05) (Figure 3A and Figure S2). Next, we systematically evaluated the impact of luteolin on four key stages: attachment, internalization, replication, and release. As shown in Figure 3, luteolin treatment caused specific inhibition at distinct stage: During attachment stage, E2 protein and viral mRNA showed no notable changes versus infected controls (Figure 3B and Figure S3); In internalization stage, both E2 and mRNA levels were significantly reduced (* *p* < 0.05) (Figure 3C and Figure S4); During replication stage, luteolin induced profound suppression of E2 and mRNA (*** *p* < 0.001) (Figure 3D and Figure S5); For release stage, no inhibitory effect was observed on viral egress or E2 expression (Figure 3E and Figure S6).

### 3.3. Luteolin Suppresses BVDV-Induced Inflammatory Pathways and Cytokine Production

**Figure 4:**
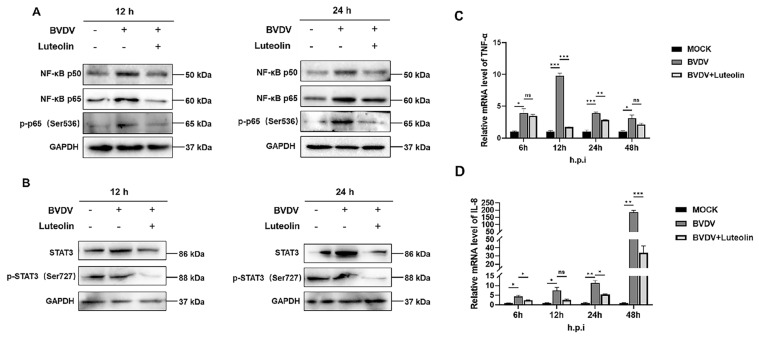
(**A**) Effect of luteolin on proteins in the NF-κB pathway after BVDV infection. (**B**) Effect of luteolin on proteins in the STAT3 pathway after BVDV infection. (**C**) Transcription level of TNF-α. (**D**) Transcription level of IL-8. (The data are expressed as the mean ± standard deviation, *** *p* < 0.001, ns: *p* > 0.05, * *p* < 0.05, ** *p* < 0.01).

Previous studies have shown that BVDV infection upregulates STAT3 expression in MDBK cells [25]. NLRP3 Inflammasome Involved with Viral Replication in Cytopathic NADL BVDV Infection and IFI16 Inflammasome Connected with IL-1β Release in Non-Cytopathic NY-1 BVDV Infection in Bovine Macrophages [26]. To explore whether luteolin’s anti-BVDV mechanism involves regulation of inflammatory mediators, we further determined the expression of NF-κB, STAT3, and NLRP3 under BVDV infection and luteolin treatment (Figure 4A, Figures S7 and S8). BVDV infection induced the expression of STAT3 at 12 and 24 hpi; luteolin treatment effectively downregulated BVDV-triggered overexpression of total STAT3 and its phosphorylated form (*p*-STAT3 Ser727) at 12 h and 24 hpi (Figure 4B, Figures S9 and S10). At different infection phases (12 h and 24 h), where BVDV strongly induced TNF-α transcription, confirmed by qPCR analysis, luteolin treatment significantly reduced mRNA levels (Figure 4C). Significant reductions in IL-8 mRNA were observed at multiple checkpoints, including 24 h (* *p* < 0.05) and 48 h (*** *p* < 0.001) post-infection. Similarly, luteolin suppressed the BVDV induced IL-8 expression (Figure 4D).

### 3.4. Luteolin Suppresses BVDV-Induced NLRP3 Inflammasome Activation and Cytokine Maturation

**Figure 5:**
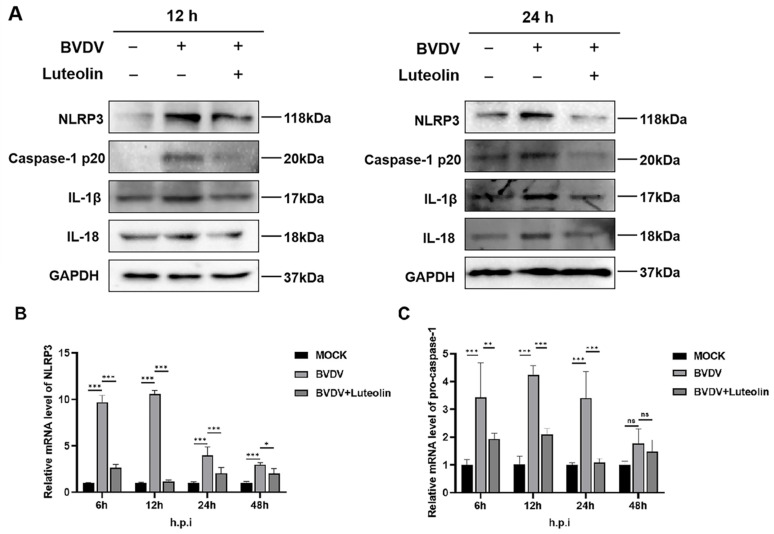
(**A**) Effects of luteolin on the expression of NLRP3 inflammasome protein after BVDV infection. (**B**) Transcription level of NLRP3. (**C**) Transcription level of pro-caspase-1. (The data are expressed as the mean ± standard deviation, *** *p* < 0.001, ns: *p* > 0.05, * *p* < 0.05, ** *p* < 0.01).

Previous study showed that cytopathic biotype BVDV infection significantly activated the NF-κB pathway and promoted the expression of NLRP3 inflammasome components (NLRP3, ASC, pro-caspase 1) as well as inflammatory cytokine pro-IL-1β in BVDV-infected bovine cells, resulting in the cleavage of pro-caspase 1 and pro-IL-1β into active form caspase 1 and IL-1β [27]. Treatment with 20 μM luteolin markedly reduced the expression of all four components, indicating disruption of inflammasome assembly and cytokine maturation (Figure 5A, Figures S11 and S12). Transcriptional repression of NLRP3 was consistently observed (Figure 5B). Q-PCR analysis results demonstrated potent inhibition of BVDV-induced NLRP3 mRNA overexpression by luteolin. Significant suppression occurred at all examined timepoints. Pro-caspase-1 mRNA expression was similarly modulated (Figure 5C). Luteolin effectively attenuated BVDV-driven transcriptional upregulation of pro-caspase-1 during active infection stages (*** *p* < 0.001), though this effect diminished at 48 hpi (*p* > 0.05). Collectively, these results demonstrate that luteolin significantly inhibits BVDV-triggered NLRP3 inflammasome activation, as evidenced by suppressed expression of core components and downstream effectors at both transcriptional and post-translational levels.

## 4. Discussion

Bovine viral diarrhea virus remains a persistent challenge to global livestock health despite widespread vaccination, primarily due to insufficient cross-protection against genetically diverse strains and the absence of clinically approved antivirals [28,29]. Our work identifies luteolin, a naturally occurring flavonoid derived from medicinal herbs including Chrysanthemum morifolium and Perilla frutescens, as a potent dual-functional agent against BVDV. It exhibits dose-dependent antiviral activity while maintaining over 90% host cell viability at therapeutic concentrations up to 20 μM, a critical advantage over conventional drugs with higher cytotoxicity profiles. Direct antiviral activity against BVDV is a well-documented property of flavonoids, with several subclasses demonstrating targeted interference with viral lifecycle processes. For instance, total flavonoids extracted from *Ammopiptanthus mongolicus* seeds have been shown to inhibit BVDV replication in MDBK cells in a concentration-dependent manner, with maximal virus inhibition rates (>80%) achieved at non-cytotoxic concentrations (<0.188 mg/mL). Mechanistically, these flavonoids preferentially act during the early stages of infection (viral entry and initial replication), as co-treatment with the virus yielded superior cell protection compared to pre- or post-infection administration—consistent with our observation that luteolin targets BVDV internalization and replication stages. Similarly, baicalein, a flavone isolated from Scutellaria baicalensis, suppresses BVDV replication by binding to the viral NS5B RNA-dependent RNA polymerase (RdRp), a key enzyme for viral genome synthesis. Molecular docking studies revealed that baicalein interacts with conserved amino acid residues in the NS5B active site, interfering with nucleotide binding and polymerase activity, mirroring our finding that luteolin reduces NS5B functionality. This convergence highlights that targeting viral replicative enzymes is a conserved antiviral strategy among flavonoids against BVDV.

Mechanistically, the CCK-8 assay confirmed that luteolin exerted no cytotoxicity on MDBK cells at concentrations ranging from 1 to 20 μM (cell viability > 90%) while inhibiting BVDV replication in a dose-dependent manner; notably, at 20 μM, it significantly reduced viral RNA copy numbers and almost completely suppressed the expression of the viral structural protein E2, directly validating its safety and efficacy as a natural anti-BVDV agent. Further dissection of the viral life cycle revealed that luteolin lacks direct virucidal activity and does not interfere with viral attachment, but potently targets the viral internalization (E2 protein and mRNA levels decreased, * *p* < 0.05) and replication stages (E2 protein and mRNA levels decreased drastically, *** *p* < 0.001)—a finding that precisely verifies the hypothesis that “luteolin exerts its anti-BVDV effect by interfering with the viral life cycle”. Complementing its direct antiviral activity, Western blot and qPCR analyses demonstrated that luteolin mitigates BVDV-induced immunopathology by inhibiting the activation of NF-κB (with reduced expression of p50, p65, and phosphorylated p65) and STAT3 (with decreased levels of total STAT3 and phosphorylated STAT3 Ser727). Concomitantly, luteolin downregulates the expression of core components of the NLRP3 inflammasome (NLRP3, pro-caspase-1), reduces caspase-1 cleavage (p20), and impairs the maturation of IL-1β and IL-18, thereby decreasing the production of pro-inflammatory factors such as TNF-α and IL-8. This dual mechanism of action fully validates the hypothesis that “luteolin inhibits BVDV-associated inflammation by regulating the NF-κB/STAT3-NLRP3 pathway” and addresses the critical issue of immunopathological damage triggered by BVDV infection.

Notably, the most significant inhibitory effect of luteolin on BVDV occurs during the viral internalization and replication stages. Therefore, these results indicate that luteolin impairs BVDV replication primarily by targeting the processes subsequent to viral attachment, contrasting with its established late-stage inhibition of enterovirus RNA synthesis or herpesviral promoter blockade [30,31]. This stage specificity implicates potential interference with host factors governing clathrin-mediated endocytosis or viral replicase complex assembly, exemplified by NS5B polymerase functionality. The M protein of the Lyssavirus genus competitively binds to NEK7 through the conserved serine at position 158, further inhibiting MAVS oligomerization, and indirectly blocks the NLRP3 initiation stage by inhibiting NF-κB, forming a “double-insurance” mechanism for immune escape [32]. Our study systematically elucidates luteolin’s capacity to disrupt BVDV-driven inflammatory cascades by concurrently targeting the NF-κB/STAT3 axis and NLRP3 inflammasome. Key findings reveal that BVDV infection within 12 h significantly upregulated expression of p65, phospho-p65 (Ser536), and p50 subunits, promoting nuclear translocation of p50-p65 heterodimers to initiate transcription of pro-inflammatory mediators (TNF-α and IL-8). However, luteolin (20 μM) significantly suppressed BVDV-induced upregulation of NF-κB signaling components at both 12 h and 24 h post-infection. Crucially, luteolin treatment suppressed both total and phosphorylated forms of these NF-κB components. Parallel inhibition was observed in the STAT3 pathway, where luteolin attenuated BVDV-induced phosphorylation of STAT3 at Ser727 (Figure 4A,B). This dual-pathway blockade translated to significant downregulation of TNF-α and IL-8 mRNA (Figure 4C,D). Previous study showed that cytopathic biotype BVDV infection significantly activated the NF-κB pathway and promoted the expression of NLRP3 inflammasome components (NLRP3, ASC, pro-caspase 1) as well as inflammatory cytokine pro-IL-1β in BVDV-infected bovine cells, resulting in the cleavage of pro-caspase 1 and pro-IL-1β into active form caspase 1 and IL-1β. Our western blot analysis revealed that BVDV infection significantly upregulated NLRP3, activated caspase-1 (p20 fragment), and mature IL-1β/IL-18 at both 12 and 24 hpi compared to uninfected controls. Luteolin effectively attenuated BVDV-driven transcriptional upregulation of pro-caspase-1 during active infection stages (*** *p* < 0.001), though this effect diminished at 48 hpi (*p* > 0.05). Collectively, these results demonstrate that luteolin significantly inhibits BVDV-triggered NLRP3 inflammasome activation, as evidenced by suppressed expression of core components and downstream effectors at both transcriptional and post-translational levels. These results demonstrate that luteolin significantly inhibits BVDV-triggered NLRP3 inflammasome activation, as evidenced by suppressed expression of core components and downstream effectors at both transcriptional and post-translational levels. Collectively, these coordinated results establish that luteolin disrupts BVDV pathogenesis through dual blockade of NF-κB/STAT3 activation and concomitant suppression of inflammatory cytokines hyperproduction. Such broad-spectrum immunomodulatory activity resonates with luteolin’s established efficacy against African swine fever virus through NF-κB/STAT3 pathway perturbation and coxsackievirus B3 via cytokine storm attenuation, further validating its utility against virally triggered inflammatory cascades [33,34]. The convergence of antiviral and anti-inflammatory actions positions luteolin as a strategic countermeasure against BVDV’s dual pathology. Its plant origin addresses emerging demands for ecologically sustainable veterinary therapeutics. Nevertheless, current limitations necessitate cautious interpretation, strain-specific effects observed require validation against prevalent subtypes; mechanistic dependencies between NF-κB/STAT3 suppression and NLRP3 inactivation should be verified using genetic knockout models; and undisclosed in vivo pharmacokinetics demand characterization. Subsequent studies must also elucidate whether luteolin inhibits BVDV-triggered pyroptosis, an immunopathological process increasingly implicated in mucosal damage. Collectively, these findings pioneer luteolin’s application against BVDV and underscore natural products as indispensable sources for next-generation antivirals targeting host-pathogen interface dynamics.

There are several challenges and improvements in the Clinical Application of luteolin against BVDV in the future. Firstly, the antiviral concentration of luteolin shown in in vitro experiments (usually ≥50 μM) far exceeds its oral bioavailability (<5%). Its poor water solubility (0.1 μg/mL) and insufficient blood drug concentration caused by the first—pass effect may weaken the in vivo antiviral effect. Nanocarrier technology (such as encapsulation with PLGA) or structural modification (glycosylation of the 7—hydroxyl group) are current improvement strategies [35]. Besides, although inhibiting the NF-κB/STAT3/NLRP3 pathway can block viral immune escape, this pathway is also involved in the host’s innate immunity. Animal models show that long-term high-dose use may lead to immunosuppression [36]. It is necessary to determine the therapeutic window through dose—gradient experiments (recommended 0.1–10 mg/kg). The RNA-dependent RNA polymerase of BVDV lacks a proof-reading function, and its high-frequency mutations may produce drug-resistant strains, posing a hidden danger of viral escape. Molecular docking shows that luteolin mainly binds to the Thr402 site of the NS5B protein, and the mutation rate in this region is as high as 10^−3^. It is recommended to use combination therapy (such as in combination with a viral protease inhibitor) to reduce the risk of drug resistance.

In conclusion, the current study demonstrated that luteolin could inhibit BVDV infection through two ways: Firstly, it directly disrupts viral internalization and RNA replication. Secondly, luteolin abrogated NF-κB/STAT3-NLRP3 axis activation by attenuating phosphorylation of p65 (Ser536) and STAT3 (Ser727), downregulating NLRP3/pro-caspase-1 transcription, and preventing caspase-1 cleavage (p20) and IL-1β/IL-18 maturation, thereby suppressing inflammatory cytokines expression. This study is the first to confirm that luteolin, a natural flavonoid compound, exerts dual antiviral and anti-inflammatory effects by blocking the internalization and replication of bovine viral diarrhea virus (BVDV) and regulating the NF-κB/STAT3-NLRP3 inflammasome pathway, providing crucial experimental evidence. Crucially, our study provides a foundation for flavonoid-based therapeutics against BVDV infection.

This study has two core limitations: first, the research model is singular, utilizing only an in vitro Madin-Darby Bovine Kidney (MDBK) cell model and a single cytopathic BVDV-1m (JLBC-28) strain, without covering non-cytopathic or other prevalent subtypes, and lacking verification in in vivo animal models, which restricts the universality and in vivo translational value of the results; second, the verification of the mechanism of action is insufficient. Although luteolin has been confirmed to regulate the NF-κB/STAT3-NLRP3 pathway and affect NS5B polymerase, the causal relationship and binding details have not been clarified through techniques such as gene knockout and molecular docking, leaving the mechanism explanation only at the correlation level.

To address the above limitations and promote translational application, the following targeted research directions are proposed: 1. Conduct in vivo verification using animal models to determine the effective therapeutic dose and administration regimen of luteolin; 2. Deepen mechanistic research to verify its binding characteristics with NS5B polymerase and the specificity of pathway regulation; 3. Optimize the administration form to improve water solubility and bioavailability through nano-delivery systems or structural modifications; 4. Establish drug-resistant BVDV strain models to explore combination therapy regimens for reducing drug resistance risks; 5. Expand virus spectrum verification to evaluate its inhibitory activity against non-cytopathic BVDV and other Flaviviridae viruses; 6. Conduct long-term toxicological studies to determine the safe therapeutic window and provide comprehensive toxicological data.

## 5. Conclusions

In conclusion, the present study demonstrates that luteolin exerts inhibitory effects on bovine viral diarrhea virus (BVDV) infection via two distinct mechanisms. First, it directly interferes with viral internalization and RNA replication processes. Second, luteolin suppresses the activation of the NF-κB/STAT3-NLRP3 signaling axis by attenuating the phosphorylation of p65 at Ser536 and STAT3 at Ser727, downregulating the transcriptional levels of NLRP3 and pro-caspase-1, and inhibiting caspase-1 cleavage into its active p20 subunit as well as the maturation of pro-inflammatory cytokines IL-1β and IL-18, thereby reducing the expression of inflammatory mediators. To our knowledge, this is the first study to verify that luteolin, a naturally occurring flavonoid, exerts dual antiviral and anti-inflammatory activities through targeting BVDV entry and replication while modulating the NF-κB/STAT3-NLRP3 inflammasome pathway. Collectively, these findings provide critical experimental evidence and lay a theoretical foundation for the development of flavonoid-based therapeutic agents against BVDV infection.

## Data Availability

The original contributions presented in this study are included in the Article/Supplementary Materials. Further inquiries can be directed to the corresponding author.

## Supplementary Materials

The following supporting information can be downloaded at: https://www.mdpi.com/article/10.3390/vetsci13010057/s1, Figures S1–S12: Western Blot original pictures.
