# Supplementary material for: Luteolin Inhibits Bovine Viral Diarrhea Virus Replication by Disrupting Viral Internalization and Replication and Interfering with the NF-κB/STAT3-NLRP3 Inflammasome Pathway
Source: Vet Sci. 2026 Jan 7;13(1):57. doi: 10.3390/vetsci13010057 (PMC12846434; doi:10.3390/vetsci13010057)
Supplement: Supplementary file 1 [file vetsci-13-00057-s001.zip › vetsci-4033785-supplementary.pdf]

Supplementary Materials

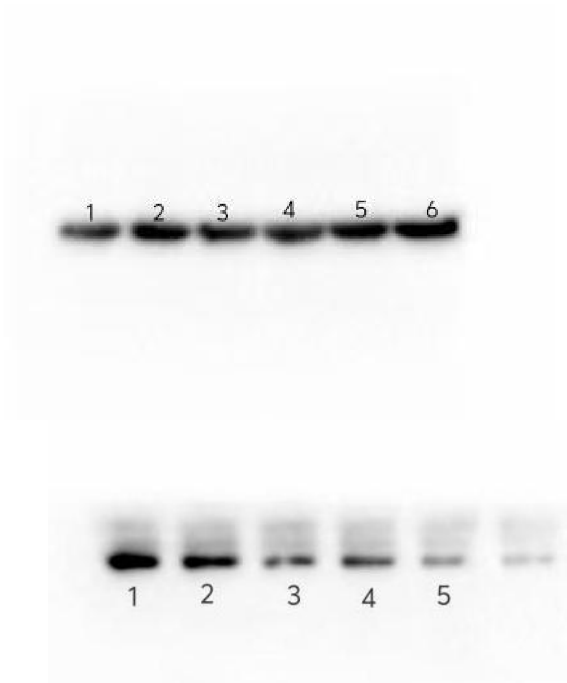

Figure S1. The picture has been cropped.

Western Blot analysis of BVDV E2 protein expression and the internal control GAPDH. Lane 1: Uninfected with BVDV and untreated with luteolin; Lane 2: Infected with BVDV and untreated with luteolin; Lane 3: Infected with BVDV + 1 μM luteolin treatment; Lane 4: Infected with BVDV + 5 μM luteolin treatment; Lane 5: Infected with BVDV + 10 μM luteolin treatment; Lane 6: Infected with BVDV + 20 μM luteolin treatment. E2 (55 kDa) is the target protein of BVDV, and GAPDH (37 kDa) serves as the internal control protein to normalize sample loading.

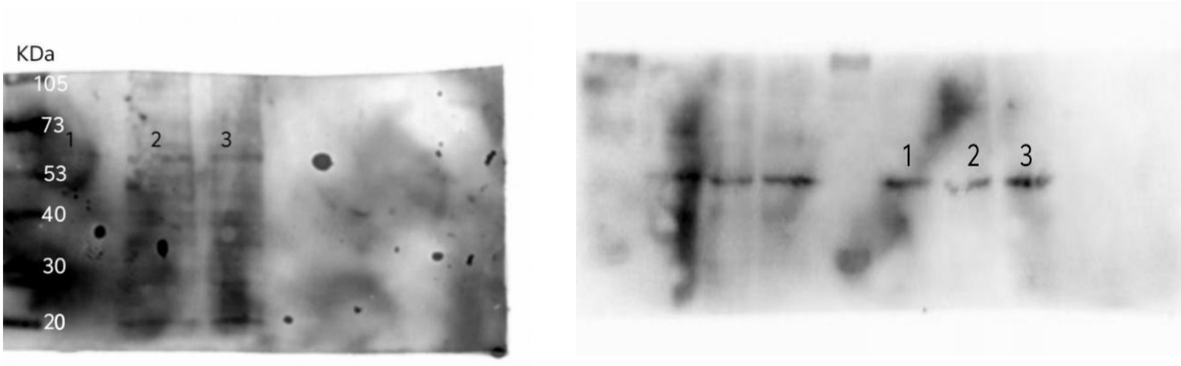

Figure S2. The picture has been cropped.

Luteolin inhibits BVDV-induced E2 protein expression in cells Western blot analysis of E2 (viral envelope protein, ~55 kDa) and GAPDH (loading control, ~37 kDa) protein levels in cells treated with: Lane 1: Control (no BVDV, no luteolin); Lane 2: BVDV infection (no luteolin); Lane 3: BVDV infection + luteolin treatment. BVDV infection (Lane 2) upregulates E2 expression relative to the control (Lane 1), while luteolin (Lane 3) attenuates BVDV-induced E2 protein accumulation. GAPDH serves as a loading control to confirm equal protein loading across lanes

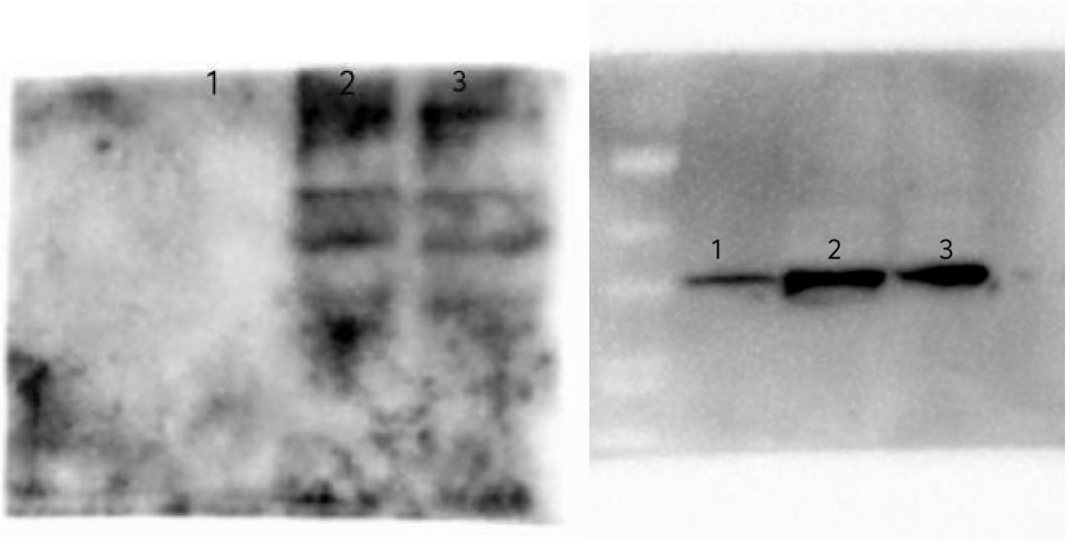

Figure S3. The picture has been cropped.

Western blot analysis of BVDV E2 protein expression during viral attachment, with GAPDH as the loading control.Lane 1: Uninfected with BVDV, no luteolin treatment (negative control) ; Lane 2: BVDV-infected (attachment stage), no luteolin treatment ; Lane 3 : BVDV-infected (attachment stage) + luteolin treatment. E2 (55 kDa) represents the BVDV envelope protein (target protein), and GAPDH (37 kDa) was used to normalize sample loading.

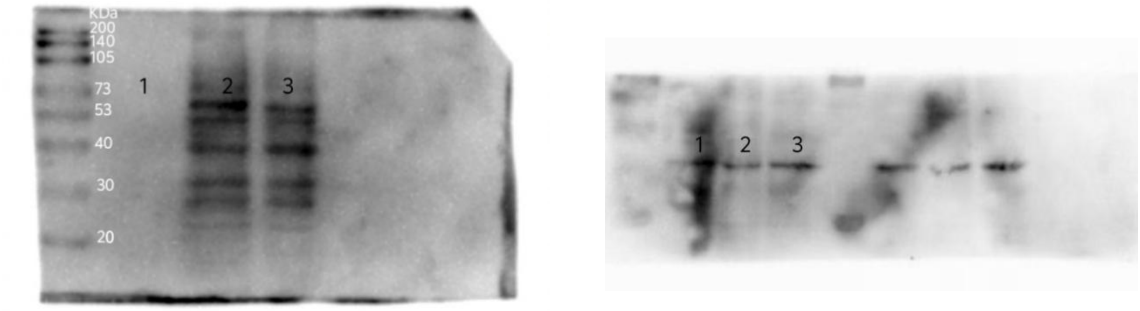

Figure S4. The picture has been cropped.

Luteolin inhibits BVDV internalization by reducing E2 protein accumulation. Western blot detection of the BVDV envelope protein E2 (~55 kDa) and the loading control GAPDH (~37 kDa) in cells treated under internalization assay conditions: Lane 1: Uninfected, untreated control ($BVDV^{-}$, $Luteolin^{-}$); Lane 2: BVDV-infected, untreated ($BVDV^{+}$, $Luteolin^{-}$); Lane 3: BVDV-infected + luteolin-treated ($BVDV^{+}$, $Luteolin^{+}$).BVDV infection (Lane 2) induces E2 protein expression relative to the control (Lane 1), while luteolin treatment (Lane 3) significantly reduces E2 levels during viral internalization. GAPDH confirms equivalent protein loading across all lanes. This result indicates that luteolin targets the internalization stage of BVDV infection to impair viral protein accumulation.

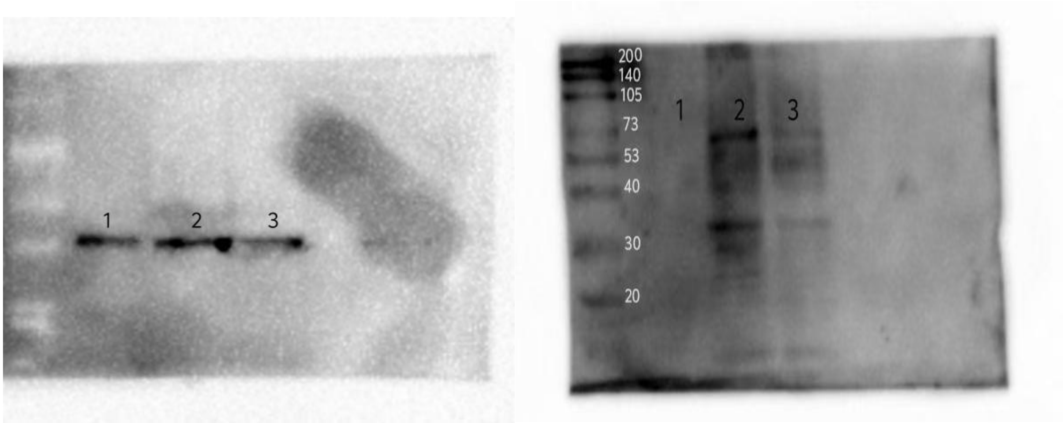

Figure S5. Luteolin attenuates BVDV replication by reducing viral E2 protein expression. Western blot analysis of the BVDV envelope protein E2 (~55 kDa) and loading control GAPDH (~37 kDa) in cells under replication assay conditions: Lane 1: Uninfected, untreated control (BVDV^{-}, Luteolin^{-}); Lane 2: BVDV-infected, untreated (BVDV^{+}, Luteolin^{-}); Lane 3: BVDV-infected + luteolin-treated (BVDV^{+}, Luteolin^{+}). BVDV infection (Lane 2) induces robust E2 protein accumulation relative to the control (Lane 1), while luteolin treatment (Lane 3) markedly decreases E2 levels during viral replication. GAPDH confirms consistent protein loading across lanes. This result demonstrates that luteolin targets the replication stage of BVDV to suppress viral protein synthesis.

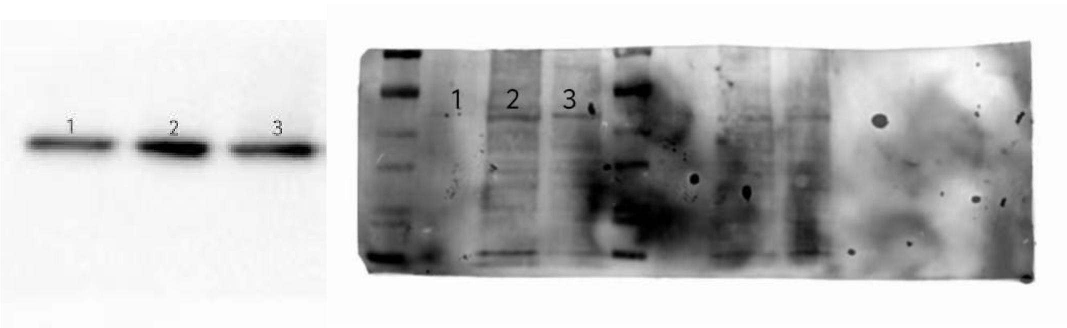

Figure S6. The picture has been cropped.

Luteolin inhibits BVDV Release (E2 Protein Detection) Experimental Groups (left to right): Lane 1 MOCK group: No BVDV infection (-) + No Luteolin treatment (-); Lane 2 BVDV group: BVDV infection (+) + No Luteolin treatment (-); Lane 3 BVDV + Luteolin group: BVDV infection (+) + Luteolin treatment (+). Western Blot (WB) Targets & Molecular Weights: E2: BVDV envelope glycoprotein (55 kDa; used as a marker for BVDV particle release); GAPDH: Loading control (37 kDa; ensures consistent sample loading).

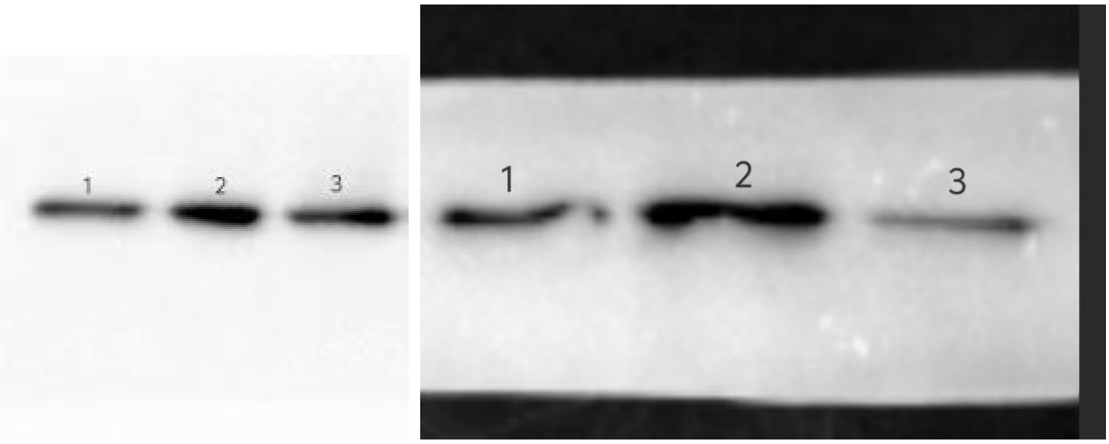

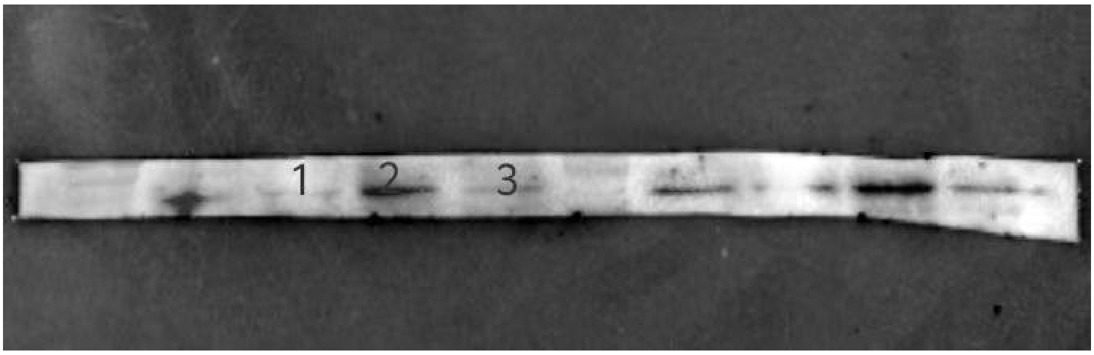

Figure S7. The picture has been cropped.

This WB examines the effect of BVDV (Bovine Viral Diarrhea Virus) infection and Luteolin (a flavonoid) treatment (at 12 hours post-treatment) on the expression/phosphorylation of NF-κB family proteins (p50, p65, and phosphorylated p65 at Ser536). GAPDH serves as the loading control (to ensure equal protein input across lanes).The 3 lanes represent: Lane 1: No BVDV infection (-) + No Luteolin treatment (-); Lane 2: BVDV infection (+) + No Luteolin treatment (-); Lane 3 BVDV + Luteolin group: BVDV infection (+) + Luteolin treatment (+).

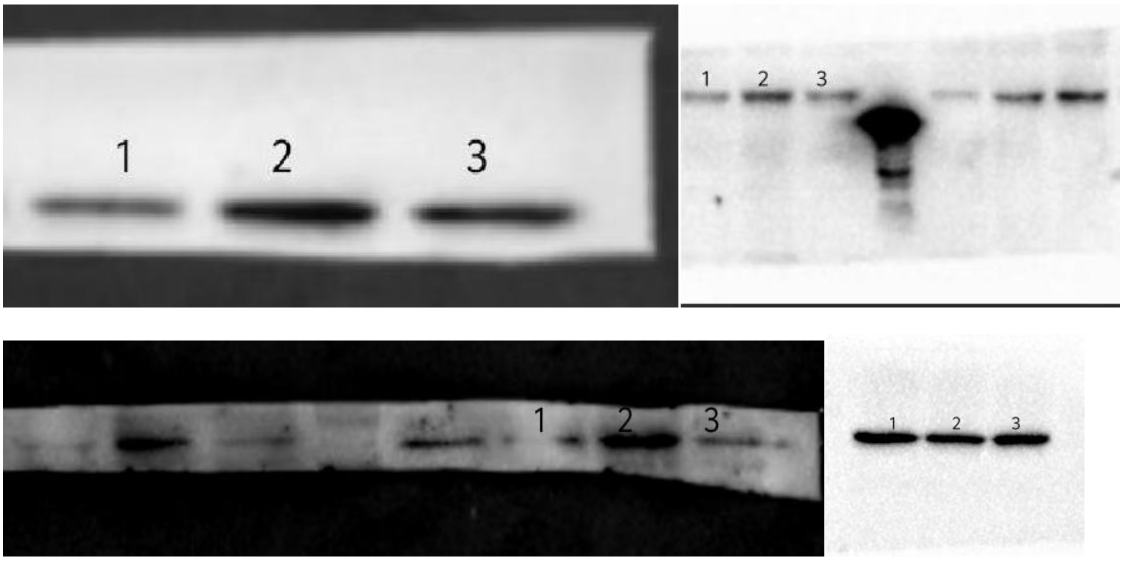

Figure S8. The picture has been cropped.

Luteolin regulates BVDV-induced NF-κB signaling activation at 24 h post-treatment Western blot analysis of NF-κB pathway proteins (p50, p65, phosphorylated p65 at Ser536) and loading control GAPDH in cells treated for 24 h: Lane 1: Uninfected, untreated control (BVDV^{-}, Luteolin^{-}); Lane 2: BVDV-infected, untreated (BVDV^{+}, Luteolin^{-}); Lane 3: BVDV-infected + luteolin-treated (BVDV^{+}, Luteolin^{+}). NF-κB p50 (50 kDa): BVDV infection (Lane 2) upregulates p50 expression relative to control (Lane 1); luteolin (Lane 3) attenuates this increase. NF-κB p65 (60 kDa): BVDV infection (Lane 2) induces p65 accumulation; luteolin (Lane 3) reduces p65 levels. p-p65 (Ser536, 65 kDa): BVDV infection (Lane 2) significantly enhances p65 phosphorylation (a marker of NF-κB activation); luteolin (Lane 3) suppresses this phosphorylation. GAPDH (37 kDa): Confirms equal protein loading across lanes.These results indicate that luteolin inhibits BVDV-induced NF-κB pathway activation by reducing both the expression and phosphorylation of NF-κB subunits.

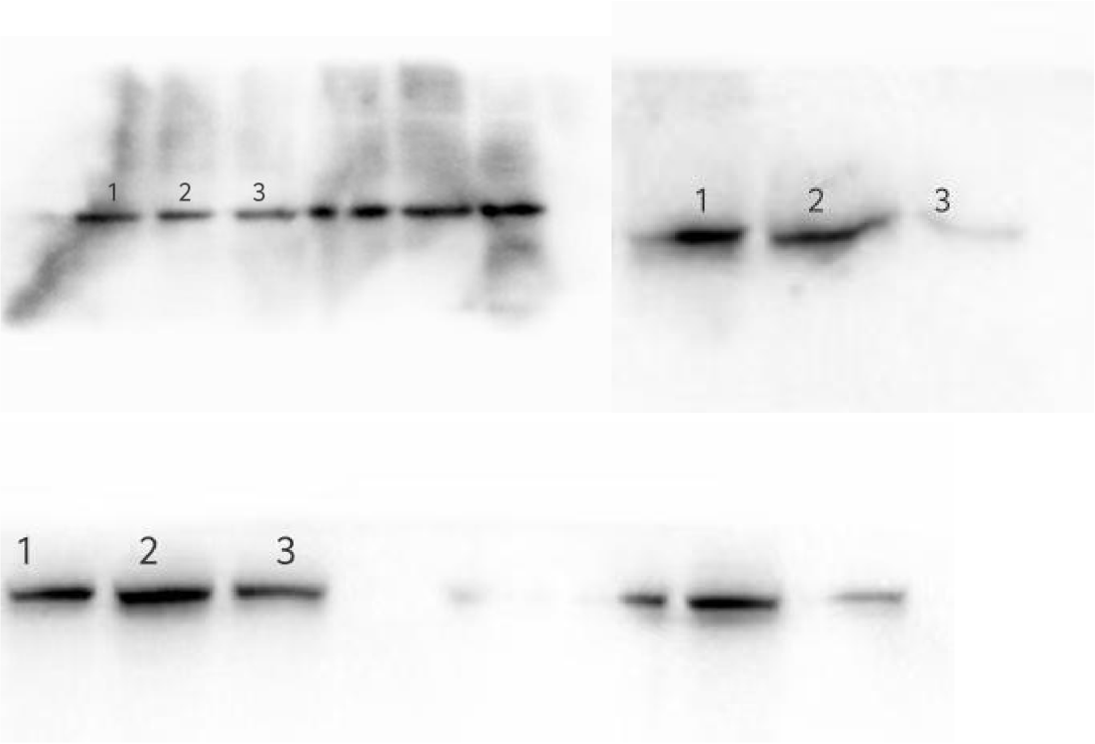

Figure S9. The picture has been cropped.

Luteolin Suppresses BVDV-Induced Activation of STAT3 Signaling Pathway in Infected Cells (12 h Post -Treatment). Experimental Groups (from left to right): Lane 1 Control group: Without BVDV infection (-) and without Luteolin treatment (-); Lane 2 BVDV group: With BVDV infection (+) and without Luteolin treatment (-); Lane 3 BVDV + Luteolin group: With BVDV infection (+) and with Luteolin treatment (+).Western Blot (WB) Detection Targets & Molecular Weights: STAT3: Total STAT3 protein (86 kDa); p-STAT3 (Ser727): Phosphorylated STAT3 (activated form) at Serine 727 (88 kDa); GAPDH: Glyceraldehyde-3-phosphate dehydrogenase (37 kDa), used as the loading control (internal reference) to normalize protein loading.

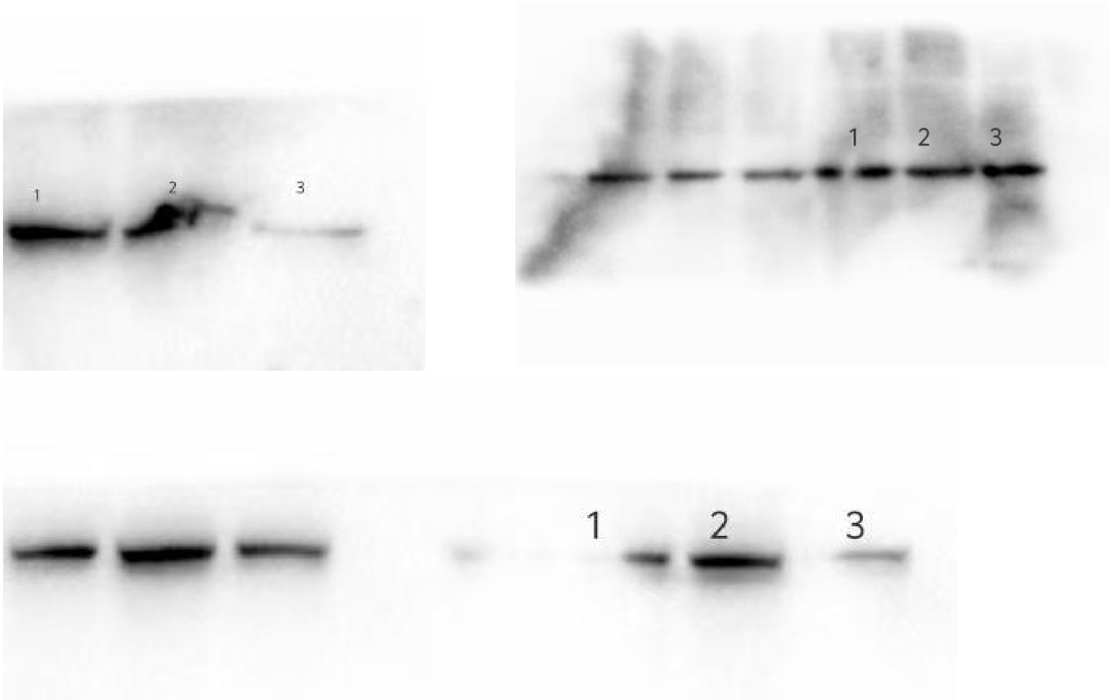

Figure S10. The picture has been cropped.

Luteolin Regulates STAT3 Signaling Pathway in BVDV-Infected Cells (24 h Post-Treatment).

Experimental Groups (left to right): Lane 1 Control group: No BVDV infection (-) + No Luteolin treatment (-); Lane 2 BVDV group: BVDV infection (+) + No Luteolin treatment (-); Lane 3 BVDV + Luteolin group: BVDV infection (+) + Luteolin treatment (+). WB Targets & Molecular Weights: STAT3: Total STAT3 protein (86 kDa); p-STAT3 (Ser727): Phosphorylated (activated) STAT3 at Ser727 (88 kDa); GAPDH: Loading control (37 kDa, normalizes protein loading).

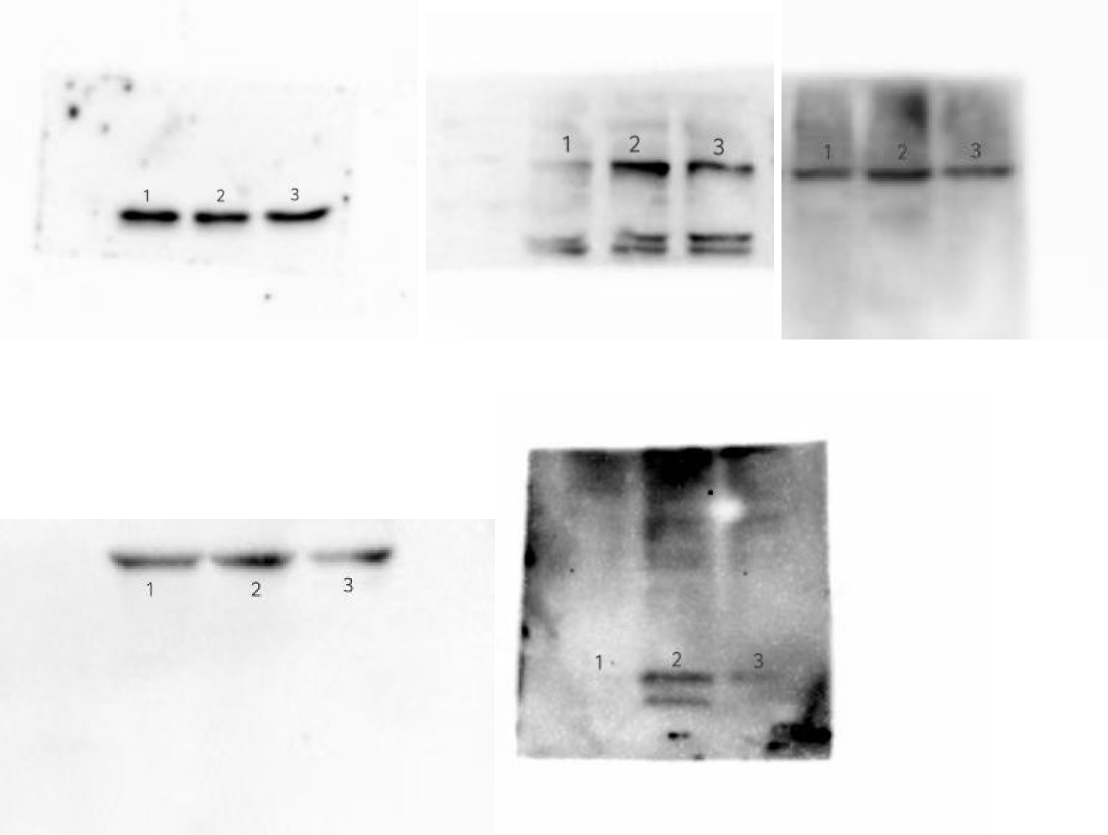

Figure S11. The picture has been cropped.

Luteolin Inhibits BVDV-Induced NLRP3 Inflammasome Activation at 12 h Post-Treatment Experimental Groups (left to right): Lane 1 MOCK group: No BVDV infection (-) + No Luteolin treatment (-); Lane 2 BVDV group: BVDV infection (+) + No Luteolin treatment (-); Lane 3 BVDV + Luteolin group: BVDV infection (+) + Luteolin treatment (+). Western Blot (WB) Targets & Molecular Weights: NLRP3: Core protein of the NLRP3 inflammasome (118 kDa); Caspase-1 p20: Activated fragment of Caspase-1 (20 kDa, indicator of inflammasome activation); IL-1β: Mature pro-inflammatory cytokine interleukin-1β (17 kDa); IL-18: Mature pro-inflammatory cytokine interleukin-18 (18 kDa); GAPDH: Loading control (37 kDa, ensures equal protein loading across groups.

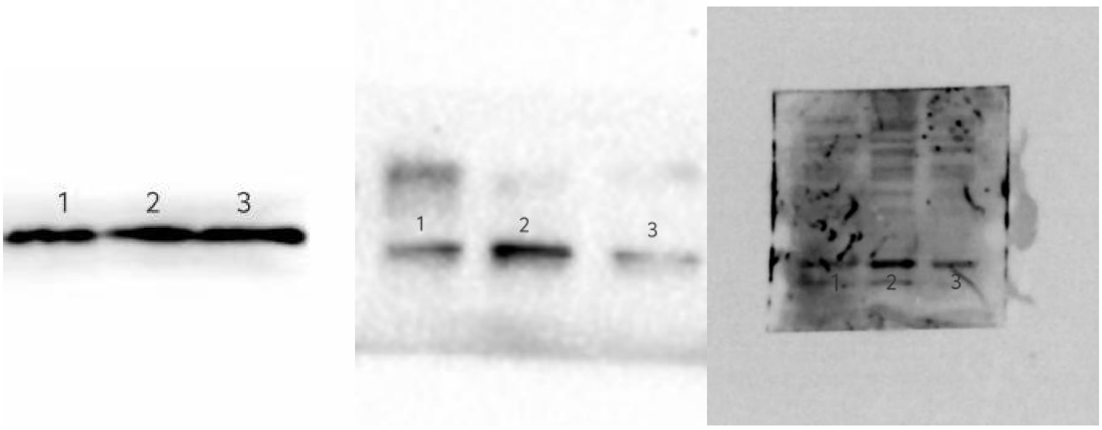

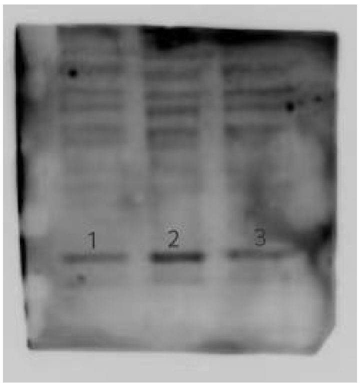

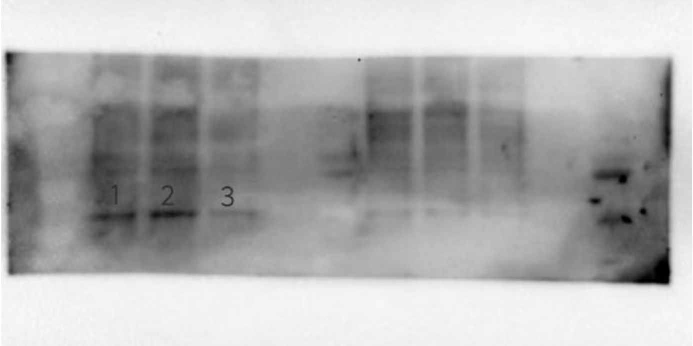

Figure S12. The picture has been cropped.

Luteolin Inhibits BVDV-Induced NLRP3 Inflammasome Activation (24 h Post-Treatment). Experimental Groups (left to right): Lane 1 MOCK (Control) group: No BVDV infection (-) + No Luteolin treatment (-); Lane 2 BVDV group: BVDV infection (+) + No Luteolin treatment (-); Lane 3 BVDV + Luteolin group: BVDV infection (+) + Luteolin treatment (+). WB Targets & Molecular Weights: NLRP3: NLRP3 inflammasome core protein (118 kDa); Caspase-1 p20: Activated fragment of Caspase-1 (20 kDa, marker of inflammasome activation); IL-1β: Mature interleukin-1β (17 kDa, downstream pro-inflammatory cytokine); IL-18: Mature interleukin-18 (18 kDa, downstream pro-inflammatory cytokine); GAPDH: Loading control (37 kDa, normalizes protein loading).
